# Prevalence of Cigarette Smoking and Associated Factors in a Large Sample of HIV-Positive Patients Receiving Antiretroviral Therapy in Vietnam

**DOI:** 10.1371/journal.pone.0118185

**Published:** 2015-02-27

**Authors:** Nhung Phuong Thi Nguyen, Bach Xuan Tran, Lu Y. Hwang, Christine M. Markham, Michael D. Swartz, Huong Thu Thi Phan, Vuong Minh Nong, Cuong Tat Nguyen, Anh Hue Nguyen, Carl A. Latkin, Damon J. Vidrine

**Affiliations:** 1 The University of Texas Health Science Center at Houston, Houston, TX 77030, United States of America; 2 Department of Behavioral Science, The University of Texas MD Anderson Cancer Center, Unit 1330, Houston, TX 77030, United States of America; 3 Institute for Preventive Medicine and Public Health, Hanoi Medical University, Hanoi, Vietnam; 4 Hanoi University of Pharmacy, Hanoi, Vietnam; 5 Authority of HIV/AIDS Control, Ministry of Health, Hanoi, Vietnam; 6 Department of Health, Behavior and Society, Johns Hopkins Bloomberg School of Public Health, Baltimore, MD, United States of America; Temple University School of Medicine, UNITED STATES

## Abstract

**Background:**

Cigarette smoking presents a salient risk for HIV-positive populations. This study is among the first to examine smoking prevalence, nicotine dependence, and associated factors in a large sample of HIV-positive patients receiving antiretroviral therapy (ART) in Vietnam.

**Methods:**

A cross-sectional study of 1133 HIV-positive people was conducted from January to September 2013 at 8 ART clinics in Hanoi (the capital) and Nam Dinh (a rural area). Smoking history and nicotine dependence (Fagerstrom Test of Nicotine Dependence–FTND) were assessed by participant self-report. Logistic regression and Tobit linear regression were performed to identify factors significantly associated with smoking outcomes.

**Results:**

Prevalence of current, former, and never smokers in the sample was 36.1%, 9.5%, and 54.4%, respectively. The current smoking proportion was higher in males (59.7%) than females (2.6%). The mean FTND score was 3.6 (SD = 2.1). Males were more likely to currently smoke than females (OR = 23.4, 95% CI = 11.6–47.3). Individuals with problem drinking (OR = 1.8, 95% CI = 1.1–2.9) and ever drug use (OR = 3.7, 95%CI = 2.5–5.7) were more likely to be current smokers. Older age and currently feeling pain were associated with lower nicotine dependence. Conversely, receiving care in Nam Dinh, greater alcohol consumption, ever drug use, and a longer smoking duration were associated with greater nicotine dependence.

**Conclusions:**

Given the high prevalence of smoking among HIV-positive patients, smoking screening and cessation support should be offered at ART clinics in Vietnam. Risk factors (i.e., substance use) linked with smoking behavior should be considered in prevention programs.

## Introduction

Emerging research has highlighted the intersection of cigarette smoking and HIV/AIDS as a significant public health concern. High prevalence of smoking among people living with HIV/AIDS (PLWHA) is well-documented in developed countries, ranging from 40% to 74% and approximately 2 to 3-fold higher than that in the general population [1–6]. Furthermore, available data indicate that smoking is an important contributor to increased morbidity and mortality [7–10], and to suboptimal effects of antiretroviral therapy (ART) in HIV-positive populations [7,11,12]. Of interest, in a large population-based Danish HIV-positive cohort, Helleberg et al. found that the number of life-years lost due to smoking (12.3 years) was substantially higher than that due to the virus (5.1 years) [9]. Additionally, HIV-positive smokers have significantly poorer immunologic response, greater risk of virologic rebound, and more frequent immunologic failure as compared to their nonsmoking counterparts [13,14].

There is a scarcity of research on this public health issue in developing countries, where both smoking and HIV epidemics have severe effects [15]. Approximately 80% of the world’s one billion smokers [16] and 95% of new HIV cases [17] are living in low- and middle-income countries. Recent findings from a sample of 657 HIV-positive individuals in China indicated a very high prevalence of current cigarette smoking (62%) [18]. Also, a study of 99 HIV-positive men in Vietnam found that 79% of the sample currently used tobacco [19]. It is unknown whether the situation of smoking among PLWHA in developing countries is the same as in developed countries. To understand the full scope of the situation, more data are needed regarding smoking prevalence and associated factors in HIV cohorts in developing countries.

Vietnam is among the countries with the highest smoking rates in the world. According to the Global Adult Tobacco Survey in 2010, approximately 19.9% adults currently smoke cigarettes in Vietnam, higher in males (39.7%) than in females (1.2%) [20]. Additionally, Vietnam has a growing HIV epidemic that initially emerged in drug using populations [21]. The estimated HIV prevalence among adults at ages 15–49 years is 0.45%, classifying the country as still in a concentrated phase. Approximately 282,787 people have contracted HIV/AIDS. Among those, 67.5% are males and 20% are at an advanced HIV/AIDS stage and requiring treatment with ART [22]. Of note, recent studies reveal that about 70% of HIV-positive individuals use illicit drugs [23] and approximately 55% use alcohol [24] in Vietnam. Moreover, cigarette smoking is considered an accepted behavior among men, but not among women in Vietnam. Given this cultural perspective and the high codependence of other substances, the smoking rate among PLWHA may be considerably higher in this developing country. To our knowledge, there has been no study about cigarette smoking, nicotine dependence, and related factors among HIV-positive people in Vietnam. Previous work on smoking has focused only on the Vietnamese general population. Therefore, this study aimed to estimate cigarette smoking prevalence, nicotine dependence, and to examine associated factors (e.g., other substance use) in a large sample of HIV-positive patients receiving ART in Vietnam. We hypothesized that cigarette smoking prevalence among HIV-positive patients was higher than that in the national general population. Our findings provide preliminary data to guide smoking prevention and cessation efforts targeting HIV-positive individuals in this developing country.

## Methods

### Study design and settings

The survey was conducted from January to September 2013 in two areas in Vietnam—Hanoi, the capital of Vietnam, and Nam Dinh, a rural area. These two cities are among the areas with the largest HIV infection burden in northern region in Vietnam, with 20,762 and 3781 PLWHA in Hanoi and Nam Dinh, respectively [21]. Five ART clinics in Hanoi and three clinics in Nam Dinh providing ART medication for HIV-positive people were included in the present study. These health centers were purposively selected based on the following criteria: 1) including national-, provincial- and district-level sites; 2) having a sufficient number of HIV/AIDS patients.

### Study subjects

Patients were approached and recruited through a convenience sampling technique. To be eligible, patients must have fulfilled the following inclusion criteria: being 18 years of age or older, inpatients or outpatients currently taking ART, and willing to provide written informed consent. Individuals who were too ill to participate in an interview were excluded from the study. Eligible patients presenting at the clinics during the study period were invited to participate in the survey until a sufficient sample size of at least 100 patients per provincial or district site, and 200 patients per national site, was obtained. The response rate was 90%. Participants were asked to complete a 30–45 minute face-to-face interview.

### Measures

#### Smoking-related variables

Based on validated smoking instruments used in previous studies [25,26], we devised 16 items to assess smoking indicators. Smoking items encompassed smoking status, age at smoking initiation, and number of cigarettes smoked per day. The primary outcome was current smoking status. Current smokers were those who had smoked at least 100 cigarettes during their lifetime and had smoked in the last 30 days at the time of interview. Former smokers were those who reported smoking at least 100 cigarettes during their lifetime but had not smoked in the last 30 days at the time of interview. Participants who had smoked less than 100 cigarettes during their lifetime were considered as never smokers. Additionally, we employed the Fagerstrom Test for Nicotine Dependence (FTND), a well-validated instrument, to evaluate the level of nicotine dependence [27]. This measure included six items, assessing the time to the first cigarette per day, number of cigarettes smoked per day, smoking in prohibited areas, most difficult cigarette of the day to give up, smoking more in the morning, and smoking if feeling ill. The items were summed to yield a total score of 0–10 with higher scores indicating higher nicotine dependence. The scores were classified by cut-points to denote levels of nicotine dependence as follows: 0–2: very low, 3–4: low, 5: moderate, 6–7: high, 8–10: very high [27].

#### Covariate variables

Sociodemographic information (i.e., age, gender, educational attainment, marital status, income, and employment status) was collected. Substance use behaviors were queried, including alcohol and illicit drug use. Alcohol consumption was measured by the brief version of the Alcohol Use Disorders Identification Test-Consumption (AUDIT-C), a brief screen for heavy drinking and/or active alcohol abuse at healthcare clinics [28]. This instrument includes three questions: 1) “How often do you have a drink containing alcohol?”; 2) “How many standard drinks containing alcohol do you have on a typical day?”; and 3) “How often do you have six or more drinks on one occasion?”. The response score for each item ranged from 0 to 4, making the total score scale range from 0 to 12. Higher scores indicated the higher likelihood patients were at risk of alcohol dependence. Threshold scores of 4 or above in men and 3 or above in women were used to identify hazardous drinkers. The Vietnamese version of AUDIT-C instrument has been validated in previous studies [24,29]. In addition, illicit drug use was examined in terms of history of drug use, type of drugs, and duration of drug use [24]. Health-related characteristics were self-reported and included weight, height, HIV-infected duration, current HIV stage, recent and nadir CD4 cell count, and ART treatment duration. Feeling pain and anxiety were assessed by two items from the EQ-5D-5L, a measure of health-related quality of life of PLWHA [30]. Accordingly, patients who answered “Slightly” to “Extremely” were considered as currently having pain or anxiety. The Vietnamese version of EQ-5D-5L instrument has been validated in a previous study [31].

### Data analyses

Descriptive statistics were conducted to compare sociodemographic, substance use behaviors, and health-related characteristics by smoking status (never, former, and current smokers). One-way analysis of variance (ANOVA) and Kruskal-Wallis tests were computed to examine differences between means of normally and non-normally distributed variables, respectively. Chi-square tests were used for categorical variables. Frequencies and distributions of smoking-related variables were also described. To test the hypothesis that cigarette smoking prevalence among PLWHA was higher than that in the general national population, we employed the one-sample z test for a binomial proportion (one-sided alternative). Multiple logistic regression and linear regression were performed to identify factors significantly associated with current smoking (current vs. non-smokers [former and never smokers combined]) and FTND score, respectively. Due to a range of [0,10], FTND scores were left- and right-censored. Censoring from above and below FTND scores did not allow us to measure exactly the values which were higher or lower than the range thresholds. Therefore, we employed a censored linear regression approach or Tobit regression to estimate linear relationships between independent variables and FTND scores [32]. Models were built based on the strategy recommended by Hosmer & Lemeshow [33]. Accordingly, any variable whose univariate test had a p-value <0.25 was a candidate for the multivariable model along with all variables of known clinical importance (e.g., alcohol and drug use). We then applied a stepwise backward model approach based on the log-likelihood ratio test including variables with a p-value<0.1. Well-documented predictors (i.e., gender, alcohol use, drug use) were kept in the final models regardless of statistical significance. Collinearity was checked by variance inflation factors. All potential interactions were examined. We assessed model calibration using Hosmer-Lemeshow goodness-of-fit test [33]. All tests of hypotheses were two-tailed with a significance level of α less than 0.05. Statistical analyses were performed with STATA version 12.0 (StataCorp LP, College Station, Texas, USA).

### Ethics statement

The use of data for this analysis was reviewed and approved by the Vietnam Authority of HIV/AIDS Control's Scientific Research Committee and the Institutional Review Board at University of Texas Health Science Center at Houston. Written informed consent was obtained from all participants after clearly introducing the survey. Respondents were able to refuse to participate or withdraw from the interview at any time, and this did not affect their continuation of health care services. Confidentiality was assured using codes of patient’s information, and secured storage was prepared for both paper questionnaires and electronic dataset.

## Results

### Participants’ characteristics

A total of 1133 HIV-positive patients participated in the survey. Among those, 1001 were from Hanoi (88.4%) and 132 were from Nam Dinh (11.6%). The mean age of the study participants was 35.5 (SD = 6.9) years old. The majority was male (58.7%), had less than a high school education (57.4%), lived with a spouse/partner (61.2%), and was currently working (79.5%). Nearly 40% of the sample reported ever using drugs. However, only 6.2% were current drug users. The rate of hazardous drinking was 18.2%. The majority of participants reported no HIV symptoms (60.8%), and a mean duration of HIV infection of 6.0 years (SD = 3.4). The average duration of ART treatment was 4.4 years (SD = 2.4). Overall, 37.7% and 44.9% of participants reported currently feeling pain and anxiety, respectively (Table 1).

**Table 1:** Characteristics of study participants by smoking status.

	Never smokers	Former Smokers	Current Smokers	Total	p-value
**N (%)**	616 (54.4)	108 (9.5)	409 (36.1)	1133 (100.0)	
**Sociodemographics**					
Age in years, mean (sd)	34.7 (7.2)	38.0 (8.0)	36.0 (5.8)	35.5 (6.9)	<0.01
Gender, n (%)					
Female	452 (73.4)	4 (3.7)	12 (2.9)	468 (41.3)	<0.01
Male	164 (26.6)	104 (96.3)	397 (97.1)	665 (58.7)	
Annual income (million VND), mean (sd)	2.7 (3.1)	3.1 (3.5)	3.0 (4.0)	2.8 (3.5)	0.25
Marital status (n, %)					
Single/Divorced/separated/widowed	268 (43.5)	36 (33.3)	136 (33.3)	440 (38.8)	<0.01
Live with spouse/partner	348 (56.5)	72 (66.7)	273 (66.8)	693 (61.2)	
Education (n, %)					
Less than high school	363 (58.9)	57 (52.8)	230 (56.2)	650 (57.4)	0.23
High school	181 (29.4)	38 (35.2)	143 (35.0)	362 (31.9)	
More than high school	72 (11.7)	13 (12.0)	36 (8.8)	121 (10.7)	
Location (n, %)					
Hanoi	553 (89.8)	94 (87.0)	354 (86.6)	1001 (88.4)	0.26
Nam Dinh	63 (10.2)	14 (13.0)	55 (13.4)	132 (11.6)	
Employment status (n, %)					
Currently working	512 (83.1)	80 (74.1)	309 (75.6)	901 (79.5)	<0.01
**Substance use, n (%)**					
Hazardous drinking	33 (5.4)	22 (20.4)	151 (36.9)	206 (18.2)	<0.01
Lifetime drug use	63 (10.2)	57 (52.8)	282 (69.0)	402 (35.5)	<0.01
Current drug use	3 (4.8)	2 (3.5)	20 (7.1)	25 (6.2)	0.51
**Health-related characteristics**					
Recent CD4 ≥ 200 cells per mm^3^, *n (%)*	390 (79.8)	61 (70.1)	236 (75.2)	687 (77.2)	0.08
BMI, mean kg/m^2^ (sd)	20.4 (2.4)	20.1 (2.4)	20.1 (2.1)	20.2 (2.3)	0.07
HIV stage, *n (%)*					
Without symptoms	263 (65.4)	44 (69.8)	149 (52.3)	456 (60.8)	
With symptoms	103 (25.6)	10 (15.9)	80 (28.1)	193 (25.7)	<0.01
AIDS	36 (9.0)	9 (14.3)	56 (19.7)	101 (13.5)	
HIV duration (years)	6.1 (3.3)	5.3 (3.1)	6.1 (3.6)	6.0 (3.4)	0.06
ART duration (years)	4.4 (2.2)	4.0 (2.1)	4.5 (2.2)	4.4 (2.4)	0.13
Currently feeling pain, *n (%)*	249 (40.4)	40 (37.0)	138 (33.7)	427 (37.7)	0.10
Currently feeling anxiety, *n (%)*	307 (49.8)	34 (31.5)	168 (41.1)	509 (44.9)	<0.01

### Smoking-related characteristics

The prevalence of current, former, and never smoking in the sample was 36.1%, 9.5%, and 54.4%, respectively. The one-sample z test for binomial proportion (one-sided alternative) with a significant p-value (p<0.01) indicated that the current smoking prevalence in our sample was significantly higher than that in the general population. Nearly half of the participants (45.6%) reported lifetime smoking (i.e., current and former smokers). Among those, only 20.9% were currently abstinent. We found significant differences in smoking status by age, gender, marital status, employment status, substance use, and current HIV stage (p<0.05). Current and former smokers were more likely to be males, older, living with a spouse/partner, and not working compared to never smokers. Of note, the proportions of both alcohol and drug use were significantly higher among current smokers than among former smokers, and lowest among never smokers (p<0.01). In addition, compared with former and never smokers, current smokers reported more severe HIV stages, with 28.1% having symptoms and 19.7% being diagnosed with AIDS. (Table 1)

The current smoking proportion was much higher in males (59.7%) than females (2.6%). Mean age at which current smokers began smoking was 17.0 years (SD = 4.1) and average duration of regular smoking was 12.6 years (SD = 8.5). The mean Fagerstrom score was 3.6 (SD = 2.1), with 34.2% at very low dependence, 33.3% at low dependence, 12.2% at moderate dependence, 15.2% at high dependence, and 5.1% at very high dependence. Nearly 27% reported smoking within 5 minutes of waking and 62.2% smoked less than 10 cigarettes per day. (Table 2)

**Table 2:** Smoking-related characteristics of current smokers (n = 409).

**Smoking-related characteristics**	
Prevalence of current smoking	
Among males	59.7%
Among females	2.6%
Age at smoking initiation, mean years (sd)	17.0 (4.1)
Number of cigarettes per day, n (%)	
< = 10	250 (62.2)
11–20	141 (35.1)
21–30	6 (1.5)
>30	5 (1.2)
Duration of regular smoking in years, mean (sd)	12.6 (8.5)
Smoke within 5 minutes of waking, n (%)	109 (26.7)
FTND score, mean (sd)	3.6 (2.1)
Nicotine dependence level, n (%)	
Very low	140 (34.2)
Low	136 (33.3)
Moderate	50 (12.2)
High	62 (15.2)
Very high	21 (5.1)

### Factors associated with current smoking

After adjusting for other variables, those found to be significantly associated with current smoking status were gender, alcohol consumption, and ever drug use. That is, HIV-positive males were 23.4 times (95% CI = 11.6–47.3,) more likely to currently smoke than HIV-positive females. Hazardous drinking (OR = 1.8, 95% CI = 1.1–2.9) and ever drug use (OR = 3.7, 95%CI = 2.5–5.7) increased the likelihood of current smoking. Additionally, participants living in Nam Dinh (OR = 1.7, 95% CI = 0.9–3.3) and having more severe HIV stages (OR = 1.5, 95%CI = 0.9–2.4; and OR = 1.8, 95% CI = 1.0–3.1) were more likely to report currently use cigarettes. These associations, however, were only marginally significant. (Table 3)

**Table 3:** Correlates of current smoking among study participants (n = 1133).

	**Univariate logistic regression**		**Multivariate logistic regression**	
	**Crude OR (95% CI)**	**p-value**	**Adjusted OR (95% CI)**	**p-value**
**Sociodemographics**				
Age in years	1.02 (1.00–1.04)	0.05		
Gender				
Female	Ref		Ref	
Male	56. 29 (31.09–101.93)	<0.01	23.44 (11.62–47.27)	<0.01
Income				
Poorest (<1.20 million VND)	Ref			
Moderate (1.21–3.00 million VND)	0.83 (0.62–1.11)	0.20		
Richest (>3.00 million VND)	1.15 (0.84–1.57)	0.38		
Marital status				
Live with spouse/partner (Yes vs. No)	1.45 (1.13–1.87)	<0.01		
Education				
Less than high school	Ref			
High school	1.19 (0.91–1.55)	0.19		
More than high school	0.77 (0.51–1.18)	0.23		
Location				
Nam Dinh vs. Hanoi	1.31 (0.90–1.89)	0.16	1.74 (0.91–3.33)	0.097
Employment status				
Currently working (Yes vs. No)	0.69 (0.51–0.92)	0.01		
**Substance use**				
Hazardous drinkers	7.12 (5.06–10.01)	<0.01	1.86 (1.16–2.97)	0.02
Ever drug users (Yes. vs. No)	11.18 (8.39–14.89)	<0.01	3.73 (2.46–5.66)	<0.01
Duration of drug use	1.02 (0.98–1.07)	0.29		
**Health-related characteristics**				
Recent CD4 (> = 200 vs. <200 cells per mm^3^)	0.84 (0.61–1.16)	0.29		
BMI category	1.00 (0.99–1.02)	0.71		
Under weight (<18.5 kg/m^2^)	0.82 (0.54–1.24)	0.34		
Normal weight (18.5–22.9 kg/m^2^)	Ref			
Overweight (> = 23 kg/m^2^)	0.63 (0.42–0.95)	0.03		
HIV stage				
Without symptoms	Ref		Ref	
With symptoms	1.46 (1.09–2.93)	0.03	1.51 (0.93–2.44)	0.09
AIDS	2.56 (1.65–3.98)	<0.01	1.54 (0.98–3.11)	0.06
HIV duration	1.01 (0.97–1.05)	0.56		
ART duration	1.02 (0.96–1.08)	0.47		
Currently feeling pain (Yes vs. No)	0.77 (0.59–0.99)	0.04		
Currently feeling anxiety (Yes vs. No)	0.78 (0.61–1.00)	0.05		

### Factors associated with nicotine dependence

Results from Tobit linear regression indicated that current smokers who were older (β = -0.5, p<0.01) and in pain (β = -0.4, p<0.05) reported lower FTND scores. In contrast, receiving care in Nam Dinh (β = 0.9, p<0.01), greater alcohol consumption (β = 0.2, p<0.01), ever drug use (β = 0.5, p<0.05), and a longer smoking duration (β = 0.1, p<0.01) were significantly associated with greater nicotine dependence. There was a marginally significant relationship between a higher BMI and a lower FTND score (β = -0.1, p = 0.096). (Table 4)

**Table 4:** Correlates of FTND score among current smoking participants (n = 409).

	**Univariate linear regression**		**Multivariate linear regression**	
	**Coefficient (SE)**	**p-value**	**Coefficient (SE)**	**p-value**
**Sociodemographics**				
Age (per 10 years)	-0.11 (0.18)	0.53	-0.49 (0.18)	<0.01
Gender				
Female	Ref			
Male	-0.53 (0.62)	0.39		
Income				
Poorest (<1.20 million VND)	Ref			
Moderate (1.21–3.00 million VND)	-0.16 (0.25)	0.53		
Richest (>3.00 million VND)	0.03 (0.26)	0.91		
Marital status				
Live with spouse/partner (Yes vs. No)	-0.18 (0.22)	0.42		
Education				
Less than high school	Ref			
High school	-0.08 (0.22)	0.73		
More than high school	-0.83 (0.38)	0.03		
Location				
Nam Dinh vs. Hanoi	0.73 (0.31)	0.02	0.89 (0.28)	<0.01
Employment status				
Currently working (Yes vs. No)	-0.31 (0.24)	0.20		
**Substance use**				
AUDIT-C score	0.17 (0.04)	<0.01	0.16 (0.03)	<0.01
Ever drug users (Yes. vs. No)	0.75 (0.22)	<0.01	0.51 (0.21)	0.02
Duration of drug use	0.04 (0.03)	0.11		
**Health-related characteristics**				
Recent CD4 (> = 200 vs. <200 cells per mm^3^)	0.30 (0.27)	0.27		
BMI	-0.12 (0.05)	0.01	-0.08 (0.05)	0.096
HIV stage				
Without symptoms	Ref			
With symptoms	-0.02 (0.30)	0.95		
AIDS	0.64 (0.34)	0.06		
HIV duration	0.00 (0.03)	0.90		
ART duration	-0.01 (0.05)	0.90		
Currently feeling pain (Yes vs. No)	-0.16 (0.22)	0.46	-0.41 (0.21)	0.047
Currently feeling anxiety (Yes vs. No)	0.26 (0.21)	0.23		
**Smoking-related variables**				
Regular smoking duration	0.07 (0.01)	<0.01	0.07 (0.01)	<0.01

## Discussion

Our study contributes to the literature by adding data on smoking status among PLWHA in a developing country, where the HIV epidemic is largely driven by drug use [21]. This survey revealed a high prevalence of smoking among HIV-positive patients receiving ART in Vietnam. More than one third of the sample (36.1%) was currently smoking, which was approximately 1.8 times higher than the national rate (19.9%) [20]. Possible explanations for disproportionately high rates of cigarette smoking among HIV-positive populations could be common factors associated with both smoking and HIV/AIDS, for instance, low socioeconomic status, substance use, and mental disorders [7]. More importantly, as the survival of PLWHA is prolonged with the use of ART, this vulnerable group is at an elevated risk of chronic smoking-related comorbidities (e.g., cardiovascular diseases, non-AIDS cancers) [7,11]. Additionally, daily tobacco use decreased the immune and virological response to ART by 40% [34]. Given the burden of cigarette smoking and its adverse health outcomes among HIV-positive patients, screening for smoking and support to quit should be integrated into current HIV/AIDS treatment in Vietnam.

Moreover, we observed a large discrepancy in current smoking rates by gender, which was dramatically higher among HIV-positive males than females (59.7% vs. 2.6%, respectively). This finding is consistent with the gender-specific rates in the Vietnamese general population (39.7% vs. 1.2%, respectively) and also in the line with previous studies among HIV samples in China and West African countries [18,35]. The striking differences in smoking rates between two genders may be due to different motivations to smoke (i.e., higher substance-codependence in male) and social contexts (i.e., smoking is culturally accepted behavior among males) in developing countries. However, the current smoking rate among HIV-positive females in our sample was nearly double the rate among the general female population (1.4%). Therefore, future interventions should take into account not only males but also females with HIV infection to reduce smoking rates in both genders. Furthermore, differences in smoking triggers between genders need to be considered in smoking cessation programs.

Of note, only 20.9% of ever smokers in our sample were currently abstinent. This estimate was lower as compared to the abstinence rate of ever smokers in the Vietnamese general population (29.3%) [20]. Previous studies have demonstrated that HIV-positive smokers face a variety of obstacles to quit smoking, including lower socioeconomic status, substance abuse, comorbid psychiatric illness, and poor access to care [36,37]. Given that achieving smoking abstinence is more difficult among PLWHA than the general population, it is critical to provide cessation support for HIV-positive smokers [38]. A recent study on smoking cessation in Vietnam found that physician brief advice was very cost-effective and should be included in the priority list of tobacco control policies [39]. Given that there are no current smoking cessation intervention programs at ART clinics in Vietnam, cessation counseling may be a feasible intervention in limited-resource settings and should be implemented at ART clinics to support HIV-positive smokers in changing their smoking behaviors.

Factors associated with current smoking among HIV-positive patients on ART in Vietnam were investigated in this study. We found that gender, ever drug use, and hazardous drinking were significantly associated with current smoking. Current smoking was common among heavy drinkers and ever drug users. These associations between smoking and other substances are well-documented in numerous studies [3–5,18,40,41]. Since HIV-positive smokers typically engage in alcohol and drug use, prevention programs targeting HIV patients should include codependence treatment for these substances.

This study also assessed factors associated with nicotine dependence among current smokers with HIV infection. We found substance use behaviors (i.e. alcohol consumption, ever drug use), smoking duration, and living at rural sites were associated with greater nicotine dependence. These findings have important clinical implications for determining higher dependency smokers, who may need intensive cessation interventions at ART sites. Additionally, we are not aware of any available data about the association between living in rural areas and greater nicotine dependence. It may be due to lower socioeconomic characteristics among HIV-positive smokers in rural areas. Another explanation may be due to less restricted smoking environments in rural areas as compared to urban areas allow HIV-positive smokers in rural areas smoke more. More research is needed to confirm this association.

The strengths of our study include a large sample of HIV-positive patients in Vietnam, which enhances the validity and reliability of our findings. Of note, nearly 40% of our sample was female, which allowed us to estimate smoking prevalence among HIV-positive women. Furthermore, our study settings included both rural and urban areas and selected sites encompassed ART clinics across all three levels of health system (i.e. national, provincial, and district ART clinics). Importantly, validated measures (e.g. FTND, AUDIT-C) used in this study make our findings more reliable and comparable. However, several limitations should also be acknowledged. First, due to the nature of a cross-sectional design, we cannot establish temporal causal relationships between independent variables and outcomes. Second, self-reported data in this study may have been susceptible to some degree of recall and social desirability biases. Third, the generalization of our study results is limited by the convenience sampling strategy due to the confidentiality of HIV-positive patients. Accordingly, caution should be used in generalizing findings in our study to other HIV populations in the country.

## Conclusions

Given the exceedingly high prevalence of cigarette use among HIV patients, screening for smoking and support to quit for both genders should be offered at ART clinics in Vietnam to reduce smoking-related adverse health outcomes. Additionally, other health risk factors (i.e., substance use) linked with smoking behavior should be considered in prevention programs.
